# Supplementary figures and images for: Structures of transcription preinitiation complex engaged with the +1 nucleosome
Source: Nat Struct Mol Biol. 2022 Nov 21;30(2):226–32. doi: 10.1038/s41594-022-00865-w (PMC9935396; doi:10.1038/s41594-022-00865-w)

Uncropped scanned gel (cf. Figure 1c).

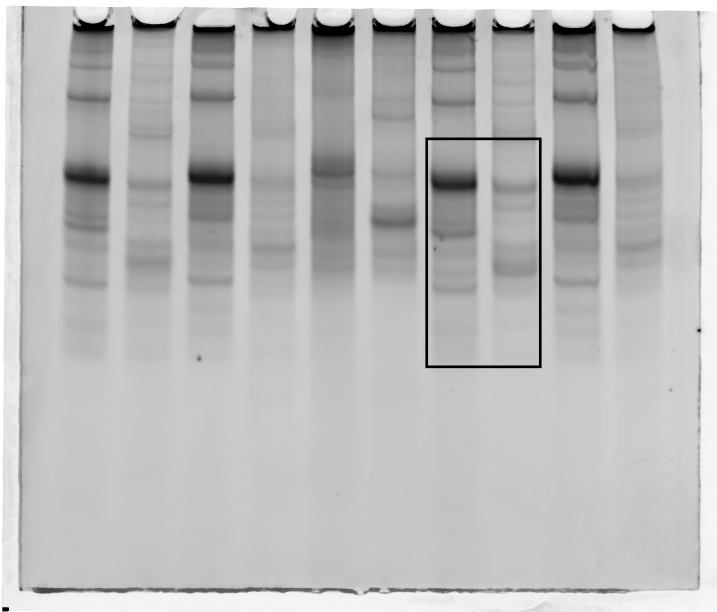

Supplement: Source Data Fig. 1 — Unprocessed gel. [file 41594_2022_865_MOESM5_ESM.pdf]
