# Supplementary material for: Structures of transcription preinitiation complex engaged with the +1 nucleosome
Source: Nat Struct Mol Biol. 2022 Nov 21;30(2):226–32. doi: 10.1038/s41594-022-00865-w (PMC9935396; doi:10.1038/s41594-022-00865-w)

Uncropped scanned gel (cf. Extended Data Figure 1a).

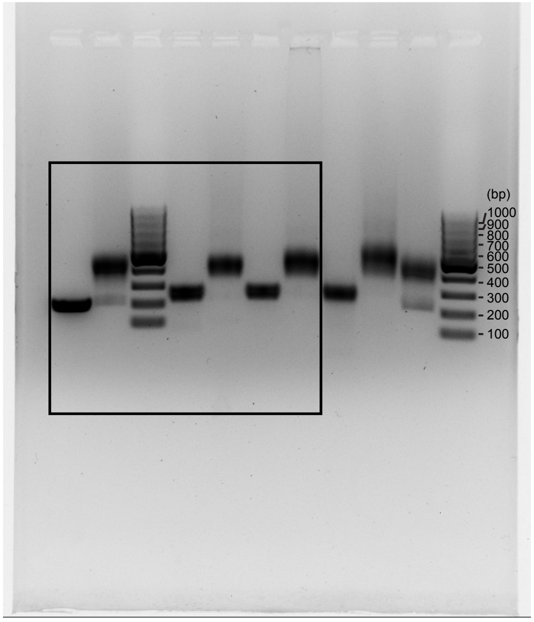

Uncropped scanned gel (cf. Extended Data Figure 1b).

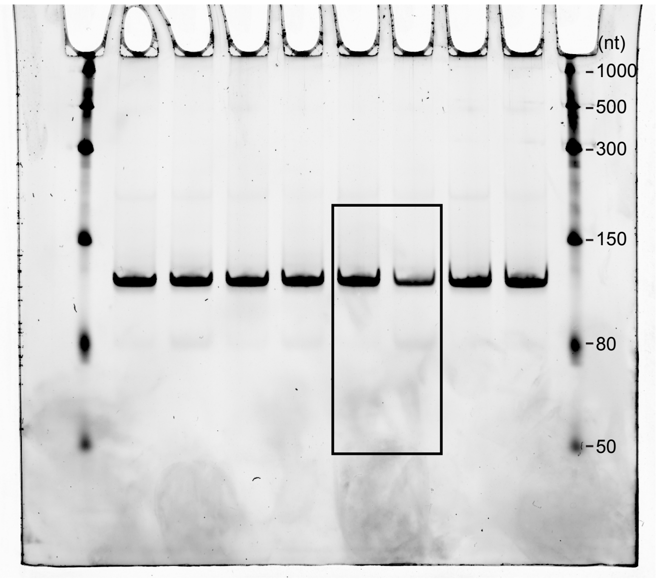

Supplement: Source Data Extended Data Fig. 1 — Unprocessed gels. [file 41594_2022_865_MOESM6_ESM.pdf]
